# Allergic proctocolitis: the clinical evolution of a transitory disease with a familial trend. Case reports

**DOI:** 10.1590/S1679-45082013000200017

**Published:** 2013

**Authors:** Ulysses Fagundes, Arnaldo José Ganc

**Affiliations:** 1 Universidade Federal de São Paulo, São Paulo, SP, Brazil.; 2 Universidade Federal de São Paulo, São Paulo, SP, Brazil; Hospital Israelita Albert Einstein, São Paulo, SP, Brazil.; Hospital Israelita Albert Einstein, Hospital Israelita Albert Einstein, São Paulo, SP, Brazil

**Keywords:** Colitis, Eosinophilia, Gastrointestinal hemorrhage, Food hypersensitivity, Infant, Case reports

## Abstract

Allergic colitis is a clinical manifestation of food allergy during the first months of life. It is estimated that genetic factors play a role in the expression of this allergic disease. This case report described the clinical progress of infants who were cousins from two distinct family groups with allergic colitis. Five infants under six months of age and of both sexes were studied, with a diagnosis of allergic colitis characterized clinically and histologically by (1) rectal bleeding; (2) exclusion of infectious causes of colitis; (3) disappearance of symptoms after elimination of cow's milk and dairy products from the child's and/or the mother's diet. Patients were submitted to the following diagnostic investigation: complete blood count; stool culture; parasitologic examination of stools; rectoscopy or colonoscopy; and rectal biopsy. Patient age varied from 40 days to six months; three were males. All patients presented with complaints of intense colic and rectal bleeding. The colonoscopy showed presence of hyperemia of the mucosa with microerosions and spontaneous bleeding upon the procedure. Microscopy revealed the existence of colitis with eosinophilia >20 e/HPF. Patients were treated with a hypoallergenic formula and showed remission of symptoms. After one year of age, all were submitted to an oral challenge with a milk formula and presented food tolerance. Allergic colitis is a disease with evident genetic inheritance and a temporary character.

## RESUMO

Colite alérgica é manifestação clínica de alergia alimentar durante os primeiros meses de vida. Estima-se que fatores genéticos exerçam papel na expressão dessa doença alérgica. Neste relato de casos, foi descrita a evolução clínica de lactentes primos entre si, pertencentes a dois grupos familiares distintos com colite alérgica. Foram estudados 5 lactentes, menores de 6 meses de idade, de ambos os gêneros, com diagnóstico de colite alérgica caracterizada clínica e histologicamente por: (1) sangramento retal; (2) exclusão de causas infecciosas de colite; (3) desaparecimento dos sintomas após eliminação do leite de vaca e derivados da dieta da criança e/ou da mãe. Os pacientes foram submetidos à seguinte investigação diagnóstica: hemograma; cultura de fezes; parasitológico de fezes; retoscopia ou colonoscopia; e biópsia retal. A idade dos pacientes variou de 40 dias a 6 meses; 3 eram do gênero masculino. Todos os pacientes apresentavam queixa de cólicas intensas e sangramento retal. A colonoscopia revelou presença de hiperemia da mucosa com microerosões e sangramento espontâneo à passagem do colonoscópio. A microscopia revelou a existência de colite com eosinofilia >20 e/CGA. Os pacientes foram tratados com fórmula hipoalergênica e apresentaram remissão dos sintomas. Após 1 ano de idade, todos foram submetidos a teste de provocação oral com fórmula láctea e apresentaram tolerância alimentar. A colite alérgica é enfermidade com evidente herança genética e de caráter temporário.

## INTRODUCTION

Food allergy is an adverse reaction to food mediated by immune mechanisms, whether IgE or not. Allergic colitis is a type of allergy that belongs to the group of non-IgE mediated food hypersensitivity, also called food-induced proctocolitis^(1)^. The pathophysiological mechanism has still not been totally identified, but it is know that it involves the presence of CD8 lymphocytes, as well as TH-2 type lymphocytes and eosinophil infiltrate in all layers of the colonic mucosa^(2)^. Additionally, circulating memory cells revealed by positive lymphocytic transformation tests suggest the involvement of T-cells in the pathogenesis of this entity, associated with secretion of tumor necrosis factor-alpha (TNF-α) by activated lymphocytes^(3)^.

It is estimated that genetic factors exert a fundamental role in the expression of this allergic disease, since a high occurrence of atopy history in families of children with eosinophilic colitis has been described^(4)^. It is estimated that the prevalence of food allergy occurs in between 2.5 and 5% of the child population, and cow's milk affects two thirds of the cases, particularly in allergic proctocolitis, 50% of cases, even in those who are being breastfed^(5)^.

In this study, we report cases of allergic colitis in infants who are cousins belonging to two distinct family groups.

## CASE REPORTS

**Figure 1:**
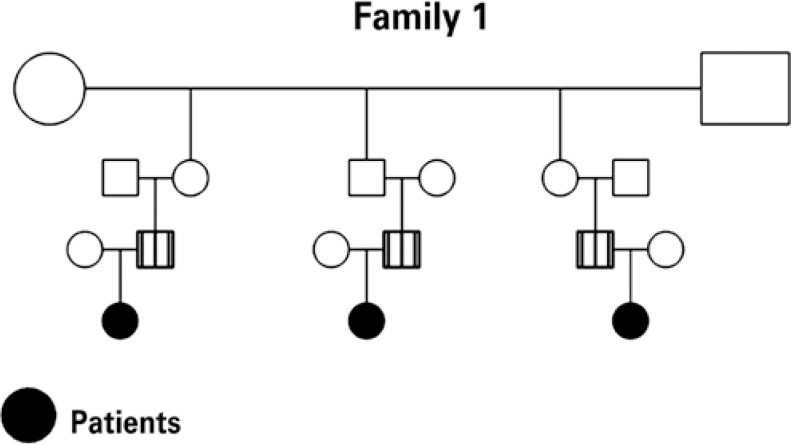
Family 1 pedigree

**Figure 2:**
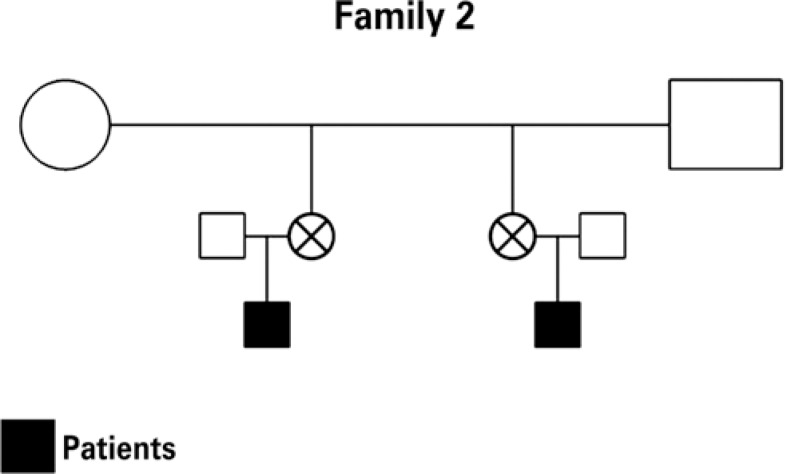
Family 2 pedigree

Five infants younger than six months of age were studied, with a mean age of four months, three of them males, belonging to two distinct family groups (Figures 1 and 2), referred to the Pediatric Gastroenterology Institute of São Paulo for investigation of the complaint of bloody stools and respective follow-up, as per a previously established investigation protocol. Four patients were receiving exclusive natural breastfeeding upon onset of symptoms, and one patient was being fed a dairy formula. The rectal bleeding manifestations occurred between 20 and 90 days of life. The initial clinical evaluation was always performed by the same professional (UFN), and included the medical history and a physical examination, as well as anthropometric measurements of weight and length.

The diagnosis of allergic colitis was characterized, clinically and histologically, as per the criteria set by Fiocchi et al.^(6)^, namely:

– presence of rectal bleeding in an infant with adequate weight and height;
– exclusion of infectious causes of colitis;
– disappearance of symptoms after elimination of cow's milk and dairy products from the child's and/or mother's diet.

Patients were submitted to the following diagnostic investigation: complete blood count; stool culture for investigation of enteropathogenic agents; stool parasitological examination of stools; rectoscopy or colonoscopy, and rectal biopsy.

The endoscopic investigation was always performed by the same professional (AG) at the Endoscopy Service of *Hospital Israelita Albert Einstein* (HIAE), in São Paulo.

Evaluation of rectal mucosal morphology was performed as per the criteria of Diaz et al.^(7)^.

Written consent was obtained from the parents for the tests.

**Figure 3:**
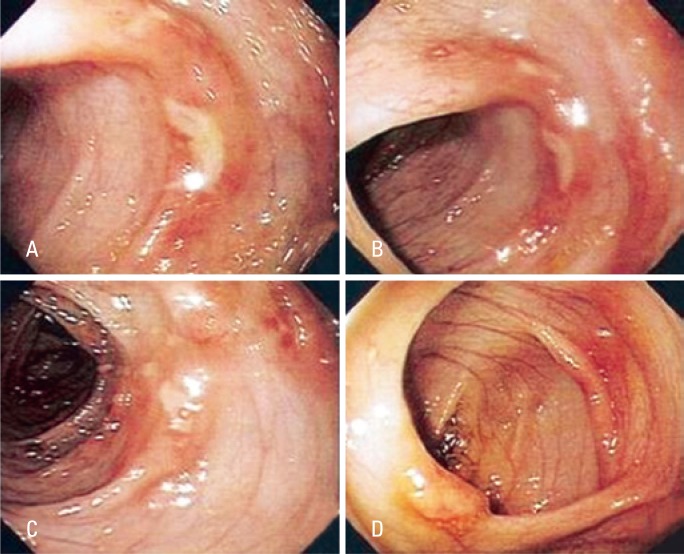
Colonic mucosa showing ulceration (A, B and D) and lymphoid nodules (C)

**Figure 4:**
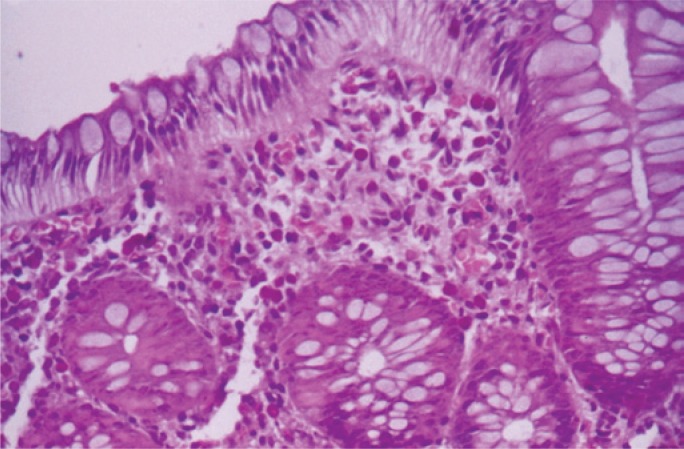
Optical microscopic photography of the colonic mucosa reveals eosinophil infiltrate in the epithelium, in lamina propria and cryptic glands

At the time of their first visit, all patients, despite the complaint of rectal bleeding, were in good clinical condition, hydrated, with no apparent condition of toxemia and presented without fever (Charts 1 and 2, and Figures 3 and 4). None of them presented with eosinophilia in the peripheral blood.

**Chart 1:** Main clinical, nutritional and laboratory characteristics of three infants of family 1

Family 1	Infant 1	Infant 2	Infant 3
Age (months)	6	4.5	1
Birth weight (g)/height (cm)	3,600/51.5	3,270/49	3,180/49
Weight (g)/height (cm) in the visit	9,615/71	6,855/66	4,695/55
Age at onset of symptoms (months)	5.5	3.5	1.2
General health status	Good	Good	Good
Proctitis	Yes	Yes	Yes
Skin paleness	No	Yes	No
Hb (g%)	11.3	11.7	-
Colonoscopy	Colitis/anal fissures and NLH	Colitis	Colitis
Pathology	Eosinophilic colitis	Eosinophilic colitis	Eosinophilic colitis
Diagnosis	Allergic colitis and proctitis	Allergic colitis and proctitis	Allergic colitis
Management	B	Amino acid formula	Hydrolyzed protein formula
Progression	Good	Good	Good
Remission (months)	16	11	12

Hb: hemoglobin; NLH: nodular lymphoid hyperplasia; B: breastfeeding.

**Chart 2:** Main clinical, nutritional and laboratory characteristics of two infants of family 2

Family 2	Infant 1	Infant 2
Age (months)	5	3.5
Birth weight (g)/height (cm)	3,165/50	3,050/48
Weight (g)/height (cm) in the visit	6,585/63	5,365/59
Age at onset of symptoms (months)	1.5	3
General health status	Good	Good
Proctitis	No	No
Skin paleness	Yes	Yes
Hb (g%)	8.5	10
Colonoscopy	NLH	Colitis
Pathology	NLH	Eosinophilic colitis
Diagnosis	Allergic colitis	Allergic colitis
Management	AAF/iron	B/AAF/iron
Progression	Good	Good
Remission (months)	18	18

Hb: hemoglobin; NLH: nodular lymphoid hyperplasia; AAF: amino acid formula; B: breastfeeding.

Due to severity of the clinical condition presented by the patients, associated with the endoscopic and anatomopathological findings that were extremely characteristic of allergic proctocolitis, it was decided to delay the challenge test due to the potential risk of intense rectal bleeding. Additionally, when submitted to a hypoallergenic diet based on extensively hydrolyzed protein formula or formula based on a mixture of amino acids, patients showed excellent clinical progress, with disappearance of rectal bleeding between 48 and 72 hours after the introduction of the hypoallergenic formula.

All patients were submitted to the challenge test with a dairy formula at ages varying from 11 to 18 months. All of them showed tolerance to the dairy formula used. The test was performed under strict medical supervision, as per Nowak-Wegrzyn et al. criteria^(8)^. At the first timepoint, they were offered 20mL of dairy formula and were observed for 45 minutes. If there was no clinical manifestation suggestive of allergy, the offer of dairy formula *ad libitum* was authorized for family members as long as they maintained close observation as to the possibility of the appearance of long-term adverse reactions.

After the triggering test with the dairy formula and the respective release for use of cow's milk and derivatives in the diet, patients were followed clinically for a period varying from 18 (minimum) to 33 (maximum) months, with an average of 26 months. During almost this entire period of follow-up, no patient presented with any clinical manifestations related to the colonic inflammatory process.

Stool culture and parasitological examination were negative in all patients.

## DISCUSSION

Allergic colitis is the most common clinical manifestation of food allergy, especially related to cow's milk protein, among infants during their first semester of life^(5)^. Rectal bleeding is the most frequent complaint, usually accompanied by intense irritability and discomfort during feeding. It has already been well established that the main allergen of the diet, during the first months of life, is cow's milk, followed by soy^(8)^, but other foods may also set off food allergy, such as milk from other mammals, eggs, wheat, fish, seafood, nuts and almonds, peanuts, and coconut. When these known allergens are a part of the diet of the nursing mother, they may be transmitted through the human milk. For this reason, infants who receive exclusive natural breastfeeding and who present with a genetic predisposition towards allergies, may also show symptoms of allergic colitis, even if not in an exacerbated manner^(4)^. Kilshaw and Cant^(9)^ demonstrated that the beta-lactoglobuline of cow's milk may be detected in samples of human milk between four and six hours after the mother has ingested cow's milk. It is important to point out that all of the five patients in this case report received natural breastfeeding during a period of their lives, and that four of them were still receiving exclusive natural breastfeeding when the signs of colitis appeared.

In the present study, it was possible to clearly demonstrate that this allergy manifestation is transitory, as has been stated by other authors^(2,4,5)^. In this study it was possible to follow up the five patients, from the initial diagnosis until the time of success after the progression time, between the 11^th^ and 18^th^ months of life of the patients. Due to severity of the clinical condition shown by the patients, associated with the extremely characteristic endoscopic and anatomopathological findings of allergic proctocolitis, we decided to postpone the challenge test, due to the potential risk of intense rectal bleeding. Additionally, when the patients were submitted to a hypoallergenic diet based on extensively hydrolyzed protein formulas or a formula based on a mixture of amino acids, there was disappearance of the rectal bleeding within 48 and 72 hours after the introduction of the hypoallergenic formula.

Currently, it is generally recognized that there is a genetic predisposition towards allergy that acts in association with one or more triggering factors^(10)^. Particularly in the case of food allergy, some of the factors that play an important role in its activation have been described, such as maternal diet, infant's diet, prematurity, absence of exclusive natural breastfeeding, deficiency of secretory IgA, deficiency of the intestinal permeability barrier^(5)^, etc. However, the occurrence of allergic colitis in family groups, such as was verified in the present study, seems to suggest strong evidence of familial genetic predisposition. Cases of allergic colitis have rarely been described among siblings or close relatives. The findings of this study confirm those of Nowak-Wegrzyn et al.^(8)^, who described a case of allergic colitis provoked by soy protein in a pair of twins, as well as those mentioned by Behjati et al.^(11)^, who described a clinical condition of allergic colitis in three brothers, within a group of 13 patients with histories of atopy in first-degree family members.

## CONCLUSION

Allergic proctocolitis manifests even in the presence of exclusive natural breastfeeding in which the predominant manifestation is the presence of hematochezia. Another characteristic is the eosinophil infiltration of the inflamed colonic mucosa, in which treatment should be made only by the use of hypoallergenic formulas with total exclusion of the protein aggressor until it can be present in the food diet. It is important to point out that in the present experience, tolerance to cow's milk protein occurred by the age of 18 months.

## References

[1] Koletzko S, Niggemann B, Arato A, Dias JA, Heuschkel R, Husby S, Mearin ML, Papadopoulou A, Ruemmele FM, Staiano A, Schäppi MG, Vandenplas Y (2012). European Society of Pediatric Gastroenterology, Hepatology, and Nutrition. Diagnostic approach and management of cow's milk protein allergy in infants and children: ESPGHAN GI Committee Practical Guidelines. J Pediatr Gastroenterol Nutr.

[2] Eigenmann PA (2009). Mechanisms of food allergy. Pediatr Allergy Immunol.

[3] Chung HL, Hwang JB, Park JJ, Kim SG (2002). Expression of transforming growth factor β1, transforming growth factor type I and II receptors, and TNF-α in the mucosa of the small intestine in infants with food protein–induced enterocolitis syndrome. J All Clin Immunol.

[4] Lake AM (2000). Food-Induced Eosinophilic Proctocolitis. J Pediatr Gastroenterol Nutr.

[5] Sicherer SH (2011). Epidemiology of food allergy. J Allergy Clin Immunol.

[6] Fiocchi A, Brozek J, Schünemann H, Bahna SL, von Berg A, Beyer K, Bozzola M, Bradsher J, Compalati E, Ebisawa M, Guzmán MA, Li H, Heine RG, Keith P, Lack G, Landi M, Martelli A, Rancé F, Sampson H, Stein A, Terracciano L, Vieths S (2010). World Allergy Organization (WAO) Special Committee on Food Allergy. World Allergy Organization: diagnosis and rationale for action against cow's milk allergy guidelines. Pediatr Allergy Immunol.

[7] Diaz NJ, Patrício FS, Fagundes U (2002). [Allergic colitis: clinical and morphological aspects in infants with rectal bleeding]. Arq Gastroenterol.

[8] Nowak-Wegrzyn A, Assa'ad AH, Bahna SL, Bock SA, Sicherer SH, Teuber SS (2009). Adverse Reactions to Food Committee of American Academy of Allergy, Asthma & Immunology. Work Group report: oral food challenge testing. J Allergy Clin Immunol.

[9] Kilshaw PJ, Cant AJ (1984). The passage of maternal dietary proteins into human breast milk. Int Arch Allergy Appl Immunol.

[10] Rothemberg ME (2004). Eosinophilic gastrointestinal disorders (EGID). J Allergy Clin Immunol.

[11] Behjati S, Zilbauer M, Heuschkel R, Philips A, Salvestrini C, Torrente F (2009). Defining eosinophilic colitis in children: insights from a retrospective case series. J Pediatr Gastroenterol Nutr.

